# Effects of Foliar Application of Iron Chlorine e6 on the Starch Physicochemical Properties and In Vitro Digestibility of Waxy Maize

**DOI:** 10.1002/fsn3.71978

**Published:** 2026-06-04

**Authors:** Linxiao Liu, Meng Zhang, Yujing Zhang, Mengyu Li, Xiangling Li

**Affiliations:** ^1^ Hebei Key Laboratory of Crop Stress Biology, College of Agronomy and Biotechnology Hebei Normal University of Science and Technology Qinhuangdao China

**Keywords:** iron chlorine e6, physicochemical properties, starch, waxy maize

## Abstract

Foliar application of iron chlorine e6 (ICE6) acts as an important agronomic practice to regulate grain quality of waxy maize. This study examined the effects of foliar application of ICe6 at six rates, 0 (T1), 22.5 (T2), 45 (T3), 67.5 (T4), 90 (T5) and 180 (T6) g ha^−1^ on the starch physicochemical properties and in vitro digestibility of waxy maize. Relative to T1, T5 and T6 significantly elevated total starch and amylopectin contents by 8.29%–8.85% and 8.39%–8.91%, respectively. Meanwhile, T4 possessed the highest starch volume‐weighted mean granule diameter. For starch granule size distribution, the proportions of medium granules increased and large granules decreased. Beyond regulating starch accumulation, ICE6 also improved starch granule morphology and surface structure, reduced relative crystallinity and gelatinization enthalpy, and enhanced pasting viscosity. Meanwhile, ICE6 decreased the content of rapidly digestible starch (RDS), increased slowly digestible starch (SDS) and resistant starch (RS). Compared with T1, T4–T6 significantly decreased RDS by 13.13%–19.13% and increased RS by 23.34%–28.65%. Mantel test, principal component analysis and random forest modeling confirmed starch granule size distribution, pasting and thermal properties as core regulators of digestibility. Overall, the optimal rate of foliar application of ICE6 was 90 g ha^−1^, which provides an important reference for improving waxy maize starch quality and developing cultivation practices for high‐quality starch production.

## Graphical abstract

In waxy maize production, foliar application of plant growth regulator iron chlorine e6 (ICE6) improves starch quality by altering its physicochemical properties and in vitro digestibility. Foliar application of ICE6 at 90 g ha^−1^ improve starch pasting properties, swelling power, starch morphology and granule size, and slowly digestible and resistant starch content, thereby enhancing structural integrity and nutritional functionality.

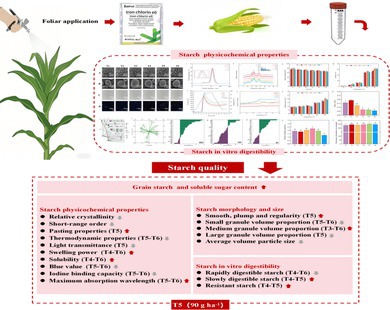

## 1 Introduction

Waxy maize (*Zea mays L. var. ceratina*) starch offers benefits including substantial output, economic efficiency and sustainable renewability (Liang et al. 2025). The cultivation area for fresh maize in China reached 1.667 million hectares, with waxy maize accounting for over 50% (Lu et al. 2025). As a major supplier of waxy maize for the Beijing–Tianjin–Hebei market, Hebei Province relies on its geographical proximity to urban agglomerations, supporting a local cultivation area of 333,000 ha. Regarding eating quality formation, amylopectin determines the glutinous texture, soluble sugars influence sweetness, and pericarp residue rate affects crispness (Li et al. 2016; Tan et al. 2020). Starch granules in waxy maize grains are almost entirely composed of amylopectin. Amylopectin constitutes the main constituent of crystalline domains, whereas amylose predominately gives rise to the amorphous domains (Buléon et al. 1998). The starch physicochemical properties are influenced by starch content, the chain length distribution of amylopectin (Jane et al. 1999), crystalline structure (Cai and Shi 2010) and particle size distribution of starch granules (Ketthaisong et al. 2013). Modifications of the physicochemical characteristics of starch can directly modulate its digestion rate (Lv et al. 2022). Based on differences in digestion rate and extent, starch is typically classified into rapidly digestible starch (RDS), slowly digestible starch (SDS), and resistant starch (RS) (Englyst et al. 1992). Starch digestion characteristics are closely linked to its nutritional quality (Zou et al. 2022), and improving nutritional quality represents a core requirement in waxy maize production.

Agricultural chemical regulation can modulate starch physicochemical properties. This approach conforms to the current demand for clean‐label food ingredients in the Beijing–Tianjin–Hebei region and fits the regional strategy of green agricultural development (Guo et al. 2023; Shi et al. 2023). Plant growth regulators are chemical substances that function similarly to natural exogenous plant hormones (Sun et al. 2021) and can modulate the entire physiological and biochemical processes in plants (Rademacher 2015; Jiang and Asami 2018). Previous studies have evaluated the regulatory effects of plant growth regulators on starch quality in maize. Appropriate concentration of coronatine can significantly enhance the activities of key enzymes in starch biosynthesis (Yu et al. 2019). Li et al. (2025) reported that exogenous 6‐benzylaminopurine alleviated drought stress in maize after anthesis and improved starch quality by regulating granule size and amylopectin content. Yan et al. (2020) found cytokinin and spermidine reduced heat damage to starch, raising grain weight and starch content. Abscisic acid optimized grain filling under high planting density (Yu et al. 2024), while brassinolide mitigated combined heat and drought stress via regulating starch multiscale structure (Huo and Yang 2022). These findings indicate that plant growth regulators can optimize starch granule structure, increase amylopectin content, thereby improving starch physicochemical properties and quality.

Iron chlorin e6 (ICE6, CAS No. 19660‐77‐6) is a novel natural plant growth regulator, it is synthesized by chelating chlorin e6 with iron ions and is initially isolated from silkworm excrement. Functionally, ICE6 can inhibit chlorophyllase activity and retard chlorophyll degradation, which further improves the plant photosynthetic efficiency (Chen 2018). Existing studies confirm that ICE6 works effectively in multiple crops (Wang et al. 2012; Xie et al. 2022; Wang, Liu, et al. 2025; Yang et al. 2025), it improves wheat yield, promotes rice growth, enhances maize lodging resistance and yield, and strengthens camellia oleifera drought tolerance. ICE6 shows considerable promise for waxy maize production in the Beijing–Tianjin–Hebei area, garnering increasing research interest. However, the regulatory effect of ICE6 on starch quality of waxy maize remains poorly understood. Therefore, this study applied different rates of ICE6 via foliar spraying to investigate its effects on starch physicochemical properties and in vitro digestibility of waxy maize. The objectives of this research were as follows: (1) to clarify the effects of foliar application of ICE6 on the starch physicochemical properties and in vitro digestion of waxy maize and (2) to determine the optimal foliar application rate of ICE6 in waxy maize production, thereby providing a theoretical basis and technical support for quality regulation of fresh‐eating waxy maize in the Beijing–Tianjin–Hebei region.

## 2 Materials and Methods

### 2.1 Experimental Materials

**FIGURE 1:**
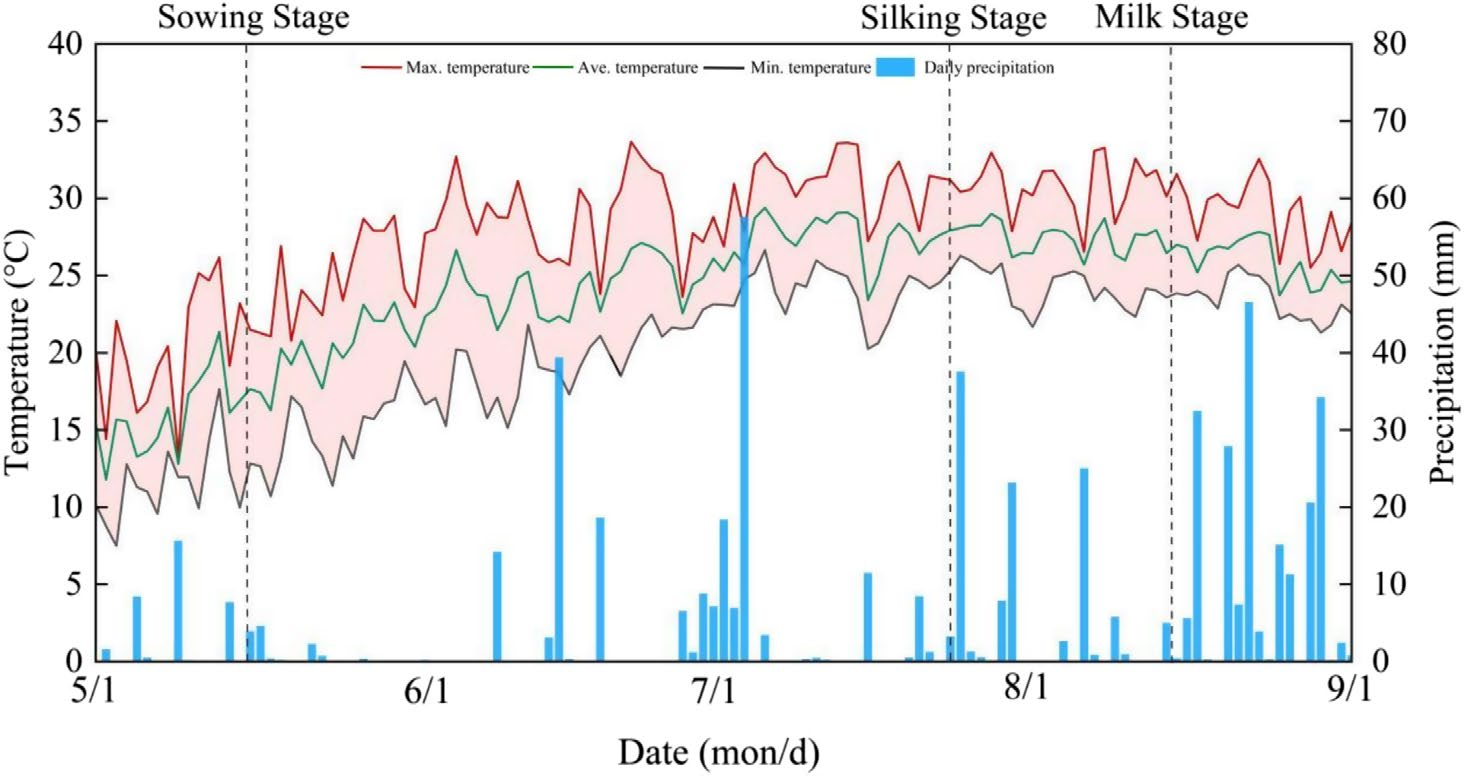
Daily temperature and precipitation during the waxy maize growing season.

This experiment was conducted from May to August 2025 in Xihenan Village, Beidaihe New District, Qinhuangdao City, Hebei Province (39.46° N, 119.22° E), which is part of the Beijing–Tianjin–Hebei coordinated development region. The site is characterized by a warm temperate, semi‐humid continental monsoon climate and a loam soil texture. The soil physicochemical properties were as follows: pH 7.04, organic matter 19.27 g kg^−1^, alkali‐hydrolysed nitrogen 84.23 mg kg^−1^, available phosphorus 94.25 mg kg^−1^, and available potassium 133.00 mg kg^−1^. The region has an average annual temperature of 18.5°C, a frost‐free period of 177 days, and an annual sunshine duration of 2745 h. Further details are provided in Figure 1.

### 2.2 Experimental Design

The experiment employed Jingkenuo 768 as the test material in a randomized block design. Six rates of ICE6 were applied, with each treatment replicated three times, resulting in a total of 18 plots. Each plot measured 64 m^2^ (8 m × 8 m), and a 120 cm wide buffer strip was established between adjacent plots. Six concentrations were set as 0, 22.5, 45, 67.5, 90, and 180 g ha^−1^, which were designated as T1, T2, T3, T4, T5, and T6, respectively. Furthermore, solutions were applied as foliar sprays using a knapsack sprayer at both the bell‐mouth stage and the flowering stage. Meanwhile, a surfactant (0.05% Tween 20) was incorporated into the spray solution, with a total application volume of 450 L ha^−1^. Specifically, applications were performed uniformly on leaf surfaces between 16:00 and 18:00 on clear, windless afternoons. In addition, a compound fertilizer (N–P_2_O_5_–K_2_O = 22–8–12) was applied at a rate of 750 kg ha^−1^ to all experimental plots. Moreover, the maize was planted with a uniform row spacing of 60 cm and a planting density of 60,000 plants ha^−1^. Finally, all other field management practices were consistent with the local high‐yield maize cultivation techniques.

### 2.3 Amylose and Amylopectin Content

At 23 days after pollination, three ears were collected from each treatment. Several kernels were excised, dried at 60°C to a constant weight, weighed, ground, and passed through a 100‐mesh sieve. Sample pretreatment followed the method described by Hansen and Møller (1975). The absorbance of the mixed standard solution was measured at wavelengths of 463, 553, 629, and 738 nm using a spectrophotometer (UV‐2600, Shimadzu, Japan). The readings were recorded as A463, A553, A629, and A738, respectively.

### 2.4 Starch Microscopic Morphology

At 23 days after pollination, three ears were collected from each treatment. Starch was extracted and prepared following the method previously reported by our research group (Lou et al. 2025). A 10 mg sample was mixed with 1 mL of deionized water in a 2 mL screw‐cap tube and vortexed to prepare a starch slurry (1% w/v). A drop of this slurry was placed on a slide with a 50% glycerol solution. The morphological characteristics of the starch were observed under both bright‐field and polarized light modes using a polarizing microscope (ECLIPSE Ci‐POL, Nikon, Japan). For each sample, 10 random fields of view were examined and imaged (Niu et al. 2025). An appropriate amount of starch granules was adhered to a sample stub, coated using an ion sputter coater, and examined under a scanning electron microscope (SU8010, HITACHI, Japan) at magnifications of 1000× and 5000× to observe the microstructure of the starch (Shi et al. 2018).

### 2.5 Starch Granule Size Distribution

The volume distribution of starch granules was determined using a laser diffraction particle size analyzer (Mastersizer 3500, Malvern, England), with ethanol employed as the dispersion medium, according to the method reported by Gu et al. (2018). Each sample was analyzed in triplicate.

### 2.6 Starch Crystal Structure

The crystal structure of the starch was determined using an X‐ray diffractometer (Bruker‐AXS D8 Advance, Karlsruhe, Germany) (Xu et al. 2019). The instrument was operated at 40 kV and 200 mA, with the diffraction angle (2*θ*) scanned from 3° to 40° at a step size of 0.04° (0.6 s per step). The relative crystallinity was calculated by analyzing the peak area and the total diffraction area using MDI Jade 6.5 software. Each sample was analyzed in triplicate.

### 2.7 Starch Infrared Spectroscopy

The molecular orderliness of starch was assessed using a fourier transform infrared spectrometer (Vertax70, Bruker, Germany) (Zhou et al. 2020). The scanning range was set to 800–1200 cm^−1^ to determine the starch surface orderliness. Each sample was analyzed in triplicate.

### 2.8 Starch Light Transmittance

Starch suspension (1% w/v) was heated in boiling water for 15 min with constant stirring. After cooling at 25°C for 60 min, the suspension was stored at 4°C. Using a UV–visible spectrophotometer (UV 2600, Shimadzu, Japan) and with distilled water (100% transmittance) as the control, the light transmittance was measured at 620 nm at 0, 24, 48, 72, 96, and 120 h (Tao et al. 2023). Each sample was analyzed in triplicate.

### 2.9 Starch Swelling Power and Solubility

160 mg sample (m0) was mixed with 8 mL of distilled water in a 10 mL centrifuge tube (m1). The mixture was prepared according to the method of Zou et al. (2020), and incubated in a shaking water bath at 50°C, 60°C, 70°C, 80°C, and 90°C for 60 min. After cooling, the mixture was centrifuged at 3000 r/min for 15 min. The precipitate was weighed (m2), and the supernatant was transferred to a centrifuge tube (m3), dried, and the total mass was recorded (m4). The starch swelling power and solubility were then calculated. Each sample analysis was performed in three replicates.

$$\text{Solubility}\;\left(\%\right)=\left(\text{m4}-m3\right)/\text{m0}\times 100\%$$

$$\text{Swelling power}\;\left(g/g\right)=m2/m0$$

### 2.10 Starch Iodine Binding Capacity, Blue Value and Maximum Absorption Wavelength

40 mg sample was mixed with 8 mL of 50 mmol L^−1^ phosphate buffer (pH 7.0). The sample was prepared following the method described by Lu and Lu (2013). The maximum absorption wavelength (*λ*_max_) of the sample was scanned from 650 to 500 nm using a UV–visible spectrophotometer (UV 2600, Shimadzu, Japan). The blue value was determined from the absorbance at 635 nm. The iodine binding capacity was calculated from the ratio of absorbances at 635 and 520 nm. Each sample was analyzed in triplicate.

### 2.11 Starch Pasting Properties

A starch sample weighing 28 g with a concentration of 7% was prepared by mixing 1.96 g of sample with 26.04 g of distilled water. The starch pasting properties were determined using a Rapid Visco Analyzer (RVA4500, Perten, Australia) following an established method (Lu et al. 2015). The relevant parameters for starch pasting properties were analyzed using the accompanying software, Thermal Cycle for Windows (TCW). Each sample was analyzed in triplicate.

### 2.12 Starch Thermal Properties

5 mg starch samples were mixed with 10 μL of distilled water, sealed in an aluminium sample pan, and equilibrated overnight in a refrigerator at 4°C. The starch thermal properties were measured using a Differential Scanning Calorimeter (DSC, Model 200 F3 Maia, NETZSCH, Bavaria, Germany) according to a published method (Lu et al. 2014). The relevant parameters for starch thermal properties were analyzed using the instrument's proprietary software. Each sample was analyzed in triplicate.

### 2.13 Starch In Vitro Starch Digestibility

The starch in vitro digestion assay was conducted following a previously established method (Li et al. 2020) with slight modifications. A 100 mg sample was mixed with 5 mL of distilled water in a 50 mL centrifuge tube and heated in a water bath at 95°C for 5 min. Subsequently, 8 mL of a mixed enzyme solution was added, and the mixture was incubated at 37°C. Aliquots of 0.1 mL were withdrawn at 0, 20, and 120 min of incubation and immediately mixed with 0.9 mL of absolute ethanol to terminate the enzymatic reaction. The resulting solutions were centrifuged at 1500 *g* for 10 min, and the glucose content in the supernatant was determined using a D‐glucose assay kit (GOPOD method). The contents of RDS, SDS, and RS were calculated according to the corresponding formulas (Wang et al. 2024). Each sample was analyzed in triplicate.

### 2.14 Data Statistical Analysis

Microsoft Excel 2021 and Origin Pro 2021 were used for data processing and plotting. SPSS 21.0 was used for analysis of variance (ANOVA). Significance analysis was performed using the least significant difference test at *p* < 0.05. The “rfPermute” and “linkET” packages in RStudio 4.5.2 were used to perform Mantel tests and create random forest models to explore the correlation between starch physicochemical properties and their in vitro digestion characteristics.

## 3 Results

### 3.1 Grain Starch Content

The soluble sugar and starch contents in grains showed an increasing trend with the increasing foliar application rate of ICE6 (Table 1). T2–T6 treatments increased the contents of soluble sugar, total starch and amylopectin compared to T1, Specifically, the total starch content significantly increased by 8.29% and 8.85% under T5 and T6, respectively, the amylopectin content increased by 8.39% and 8.91%, respectively.

**TABLE 1:** Effects of foliar application of iron chlorine e6 on the grain nutritional quality of waxy maize.

Treatment	Sugar (%)	Total starch (%)	Amylopectin (%)	Amylose (%)	Amylopectin proportion (%)
T1	5.71 ± 0.56d	42.92 ± 9.38b	42.77 ± 9.41bc	0.13 ± 0.01a	99.55 ± 0.02a
T2	6.43 ± 0.57c	43.28 ± 8.36b	43.13 ± 8.30b	0.12 ± 0.01a	99.59 ± 0.01a
T3	7.11 ± 0.42b	43.63 ± 4.23b	43.50 ± 4.36b	0.12 ± 0.01a	99.60 ± 0.03a
T4	8.40 ± 0.42a	44.23 ± 6.73b	44.07 ± 6.66b	0.12 ± 0.03a	99.59 ± 0.06a
T5	8.60 ± 0.35a	46.48 ± 2.81a	46.36 ± 2.41a	0.10 ± 0.04a	99.53 ± 0.09a
T6	8.20 ± 0.51a	46.72 ± 9.48a	46.58 ± 9.41a	0.09 ± 0.02a	99.55 ± 0.02a

*Note:* T1, T2, T3, T4, T5, and T6 denoted as the iron chlorine e6 application rates at 0, 22.5, 45, 67.5, 90, and 180 g ha^−1^, respectively. Data are means of three replications. Means with no letter in common indicate significant differences between different treatments by least significant difference test (*p* < 0.05).

### 3.2 Starch Microscopic Morphology

**FIGURE 2:**
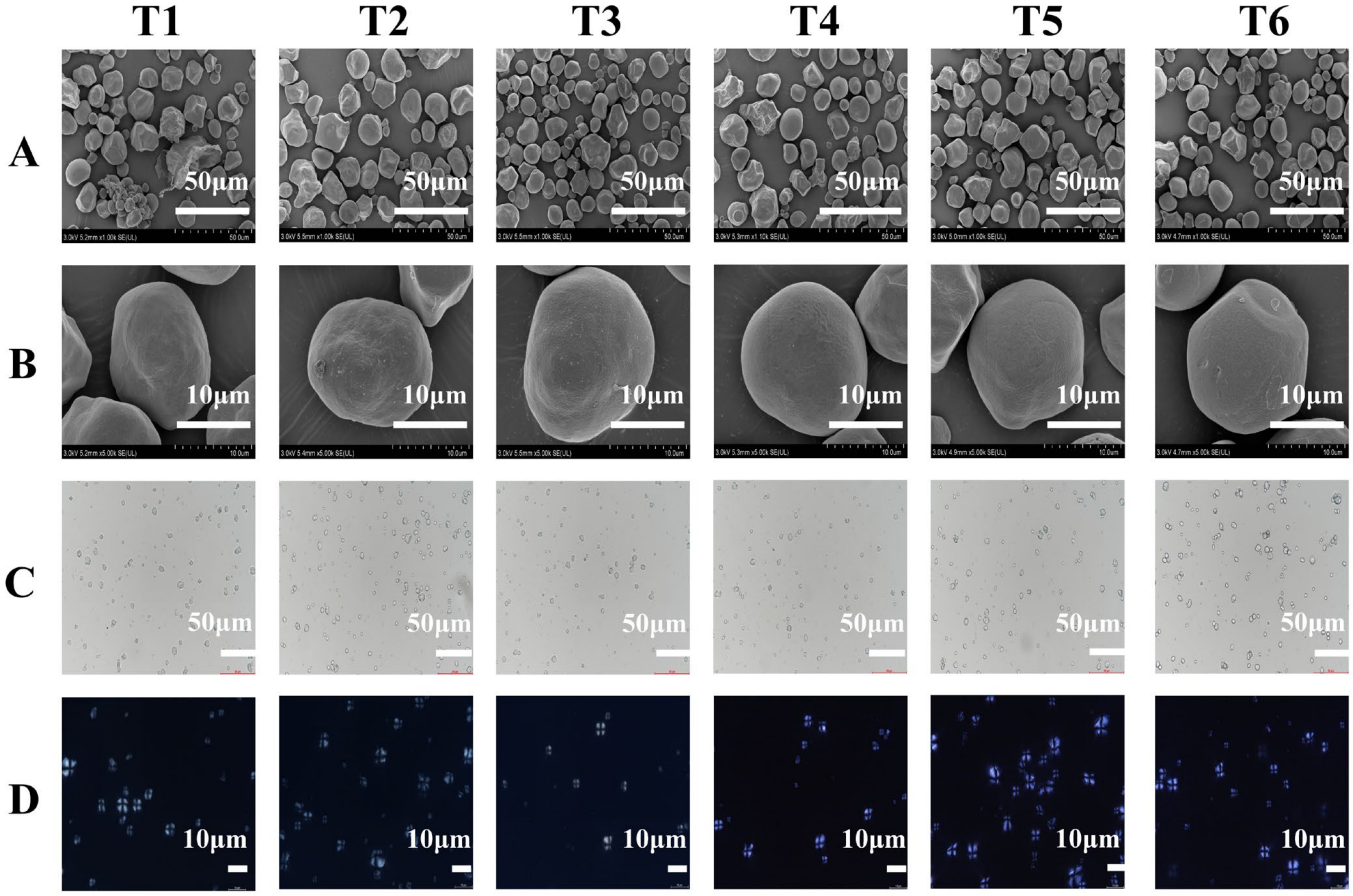
Effects of foliar application of iron chlorine e6 on the starch scanning electron microscopy (A, B), optical microstructure (C), and polarized light cross structure (D) of waxy maize.

Scanning electron microscopy observations (Figure 2A,B) revealed that starch granules across all treatments shared a consistent morphology, predominantly comprising round, oval, and polygonal forms. Differences were noted in starch granule integrity, surface condition, and particle size across all treatments. Starch granule morphology varied markedly among treatments; granules in T1 exhibited minor fractures, those in T2 were relatively small with numerous surface protrusions, those in T3 had localized surface bulges, those in T4 featured smooth surfaces and regular rounded profiles. T5 produced the largest, nearly spherical, plump granules with pristine surfaces. In contrast, T6 led to evident surface indentations on starch granules.

### 3.3 Starch Optical and Polarized Light Microscopy

Optical microscopy observations (Figure 2C) revealed variations in the dispersibility and regularity of starch granules among the different treatments. Starch granule dispersibility and morphology exhibited gradual enhancement. T1 and T2 treatments presented poor dispersion, severe agglomeration, and irregular shapes; T3–T5 treatments gradually ameliorated these features, and T6 treatment achieved the best dispersion, uniformity, and morphology. Polarized light microscopy (Figure 2D) revealed that starch granules from all treatments exhibited a “Maltese cross” extinction pattern, confirming their crystalline nature. However, differences were observed in the order of their crystalline structures. T1–T3 treatments had disordered and weak signals; T4 and T5 treatments recovered gradually, and T6 treatment showed the strongest, brightest signals with the most ordered crystalline structure.

### 3.4 Starch Particle Size Distribution

**FIGURE 3:**
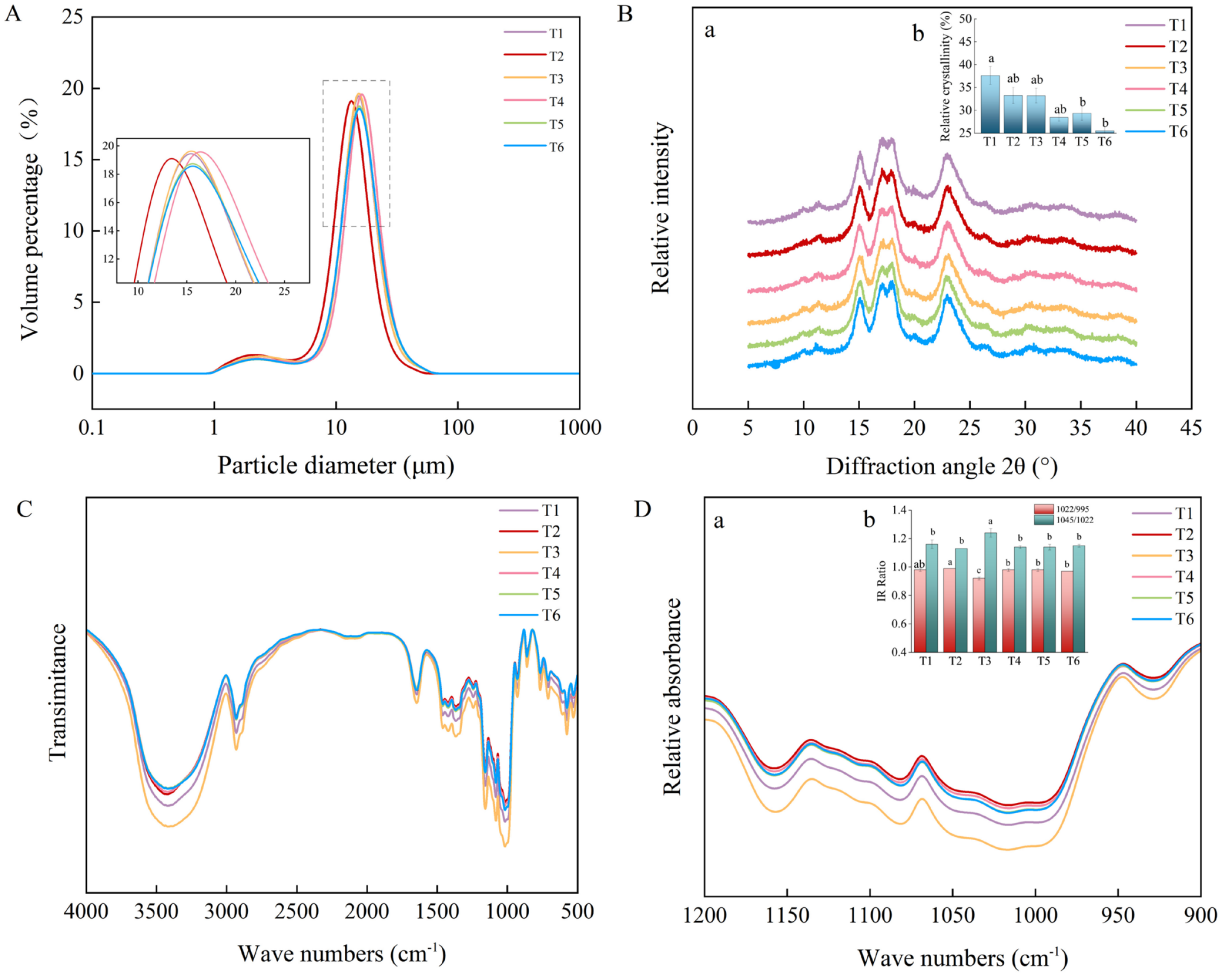
Effects of foliar application of iron chlorine e6 on the starch granule volume distribution (A), X‐ray diffraction (XRD) patterns (a) and (b) relative crystallinity (B), Fourier‐transform infrared (FTIR) full spectrum (C), and FTIR spectra in the 900–1200 cm^−1^ region (a) and IR absorbance ratios of 1022/995 and 1045/1022 cm^−1^ (b) reflecting short‐range ordered structure (D). T1, T2, T3, T4, T5, and T6 denoted as the iron chlorine e6 application rates at 0, 22.5, 45, 67.5, 90, and 180 g ha^−1^, respectively. Different lowercase letters indicate significant differences among treatments (*p* < 0.05).

The starch particle volume distribution exhibited a unimodal trend, with the peak concentrated in the 10–20 μm range (Figure 3A). As indicated in Table 2, the effects of foliar application of ICE6 on the volume distribution proportion of starch granules were observed. Compared with T1, T3 treatment significantly increased the volume proportion of small starch granules, T5 and T6 treatments significantly reduced this proportion by 13.19% and 17.50%, respectively. Meanwhile, T3–T6 treatments significantly increased the volume proportion of medium starch granules, and T4 promoted the effective conversion and accumulation of small granules into medium‐sized ones. By contrast, the volume proportion of large starch granules showed an opposite trend. T2–T5 treatments were significantly lower relative to T1. Furthermore, distinct differences were observed in the volume‐weighted average particle size across treatments. Comparison with T1, T5 presented relatively larger average particle sizes; T2–T6 showed significant reductions in particle size by 2.47%–5.87%.

**TABLE 2:** Effects of foliar application of iron chlorin e6 on the distribution and average granule diameter of waxy maize.

Treatment	Average granule diameter (μm)	Volume distribution (μm)		
		< 5 μm	5–15 μm	> 15 μm
T1	15.00 ± 0.21a	8.57 ± 0.04b	33.75 ± 0.20d	57.69 ± 0.23a
T2	14.12 ± 0.06c	8.55 ± 0.02b	35.97 ± 0.23b	55.49 ± 0.21c
T3	14.22 ± 0.02c	9.54 ± 0.21a	51.45 ± 0.35a	39.01 ± 0.19d
T4	14.21 ± 0.07c	8.53 ± 0.03b	35.35 ± 0.27c	56.12 ± 0.24b
T5	15.07 ± 0.07a	7.44 ± 0.03c	36.00 ± 0.48b	56.56 ± 0.51b
T6	14.63 ± 0.07b	7.07 ± 0.10d	35.40 ± 0.26c	57.53 ± 0.16a

*Note:* T1, T2, T3, T4, T5 and T6 denoted as the iron chlorine e6 application rates at 0, 22.5, 45, 67.5, 90 and 180 g ha^−1^, respectively. Data are means of three replications. Means with no letter in common indicate significant differences between different treatments by least significant difference test (*p* < 0.05).

### 3.5 Starch Relative Crystallinity

The X‐ray diffraction analysis results (Figure 3B) indicated that the diffraction patterns of starch from all treatments followed a consistent trend. Two strong diffraction peaks were observed at 2*θ* angles of 15° and 23°, along with a continuous double peak appearing at 17° and 18°. The starch relative crystallinity exhibited a decreasing trend with the increase of ICE spraying concentration (Figure 3B). Compared with T1, T2, T3, T4, T5, and T6 treatments showed significantly decreased by 11.56%, 11.70%, 24.30%, 22.01%, and 32.16%, respectively.

### 3.6 Starch Short‐Range Ordered Structure

The absorption peak positions in the FTIR spectra were consistent across all treatments, although differences were observed in their relative absorbance values (Figure 3C). The foliar application of ICE6 enhanced the starch surface orderliness, reducing its surface disorder (Figure 3D). The 1022/995 cm^−1^ ratio was significantly lower in T2, T4, T5, and T6 treatments than in T1; T3 treatment showed an increase relative to T1. The 1045/1022 cm^−1^ ratio significantly decreased in T3 treatment compared with T1, while no significant differences were observed.

### 3.7 Starch Pasting Properties

**FIGURE 4:**
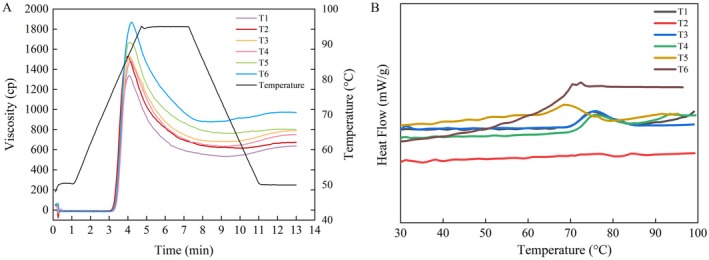
Effects of foliar application of iron chlorine e6 on the starch pasting properties (A) and thermal properties (B) of waxy maize. T1, T2, T3, T4, T5, and T6 denote the iron chlorine e6 application rates at 0, 22.5, 45, 67.5, 90, and 180 g ha^−1^, respectively.

The differences were observed in starch pasting properties indices following foliar application of ICE6 (Figure 4A and Table S1). Compared with T1, the PV of T2–T6 significantly increased by 14.30%–39.67%, TV increased by 14.82%–64.10%, FV increased by 5.75%–52.59%, and BD increased by 9.08%–23.54%, respectively. Among them, T5 and T6 treatments exhibited the most significant promoting effects on these parameters. Meanwhile, the SB in T2 and T5 showed significant decreases by 40.58% and 61.02%, respectively. Among them, T3, T4, and T6 treatments exhibited a decreasing trend, but the differences were not statistically significant.

### 3.8 Starch Thermal Properties

Foliar application of ICE6 resulted in significant differences in the thermal properties of waxy maize starch (Figure 4B and Table S2). Compared with T1, T3, and T4 treatments exerted no significant effect on the To; T5 and T6 treatments significantly decreased this parameter by 9.23% and 12.27%, respectively. Tp was significantly elevated in T2–T4 treatments, and no significant effects were observed for T5 and T6 treatments. Compared with T1, ΔH_gel_ and ΔH_ret_ showed significant reductions across all treatments. The reductions of ΔH_gel_ in T2 to T6 treatments ranged from 14.94% to 48.92%; those for ΔH_ret_ reached 29.61% to 69.83%. Meanwhile, T1 exhibited the highest relative crystallinity among all treatments.

### 3.9 Starch Light Transmittance, Swelling Power, and Solubility

**FIGURE 5:**
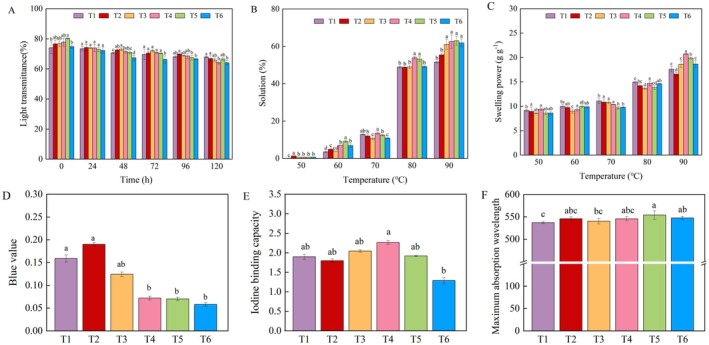
Effects of foliar application of iron chlorine e6 on the starch light transmittance (A), swelling power (B), solubility (C), blue value (D), iodine binding capacity (E), and maximum absorption wavelength (F) of waxy maize. Different lowercase letters above the bars indicate significant differences among different treatments (*p* < 0.05).

The results of starch light transmittance (Figure 5A) indicated that the light transmittance of starch showed a declining trend with prolonged storage time at 4°C storage conditions. T5 exhibited significantly higher light transmittance at 0 h; it can enhance the initial transparency of starch paste; its long‐term stability in terms of transparency is poor. As shown in Figure 5B,C, both swelling power and solubility increased gradually as temperature rose, reaching average values of 63.62 g g^−1^ and 14.11% at 90°C. Compared with T1, T2–T6 significantly increased starch swelling power by 12.71%–46.61% and solubility by 7.49%–22.17%. Among them, T4 and T5 displayed the most desirable regulatory enhancement effects.

### 3.10 Starch Blue Value, Iodine Binding Capacity and Maximum Absorption Wavelength

The starch blue value (Figure 5D), iodine binding capacity (Figure 5E), and *λ*_max_ (Figure 5F) showed variations with the increasing of ICE foliar application rate. Compared with T1, T2 treatment resulted in a significant increase of 19.50% in blue value. In contrast, T3–T6 treatments led to a significant decrease of 22.01%–63.52%. The response of starch iodine binding capacity to foliar application of ICE6 exhibited differential patterns. Among them, T3 and T4 treatments increased starch iodine binding capacity; T2, T5, and T6 treatments displayed a decreasing trend. Furthermore, the foliar application of ICE6 increased the maximum absorption wavelength, the enhancement effect was most pronounced under T5 and T6 treatments.

### 3.11 Starch In Vitro Digestion

The in vitro digestion fractions exhibited differential responses to foliar application rate of ICE6 (Table 3). Compared with T1, the RDS content in T4, T5, and T6 treatments significantly decreased by 13.13%, 19.13%, and 17.50%, respectively. The SDS content showed an increasing trend, with T2, T4, T5, and T6 treatments showing higher content relative to T1. The trend in RS content was consistent with that of SDS. Compared with T1, T4 and T5 treatments showed significant increases of 23.34% and 28.65%, respectively. Foliar application of ICE6 reduced the RDS content and increased the SDS and RS contents.

**TABLE 3:** Effects of foliar application of iron chlorine e6 on the starch in vitro digestibility of waxy maize.

Treatment	RDS (%)	SDS (%)	RS (%)
T1	53.02 ± 1.51a	19.70 ± 1.11a	27.29 ± 2.19b
T2	51.28 ± 2.30a	21.44 ± 2.66a	27.29 ± 1.33b
T3	50.70 ± 1.89a	19.70 ± 1.04a	29.64 ± 1.30ab
T4	46.06 ± 2.61b	20.28 ± 0.82a	33.66 ± 0.92a
T5	42.88 ± 1.00b	22.02 ± 2.66a	35.11 ± 2.01a
T6	43.74 ± 0.89b	20.28 ± 1.92a	32.64 ± 2.23ab

*Note:* 0 T1, T2, T3, T4, T5 and T6 denoted as the iron chlorine e6 application rates at 0, 22.5, 45, 67.5, 90 and 180 g ha^−1^, respectively. Data are means of three replications. Means with no letter in common indicate significant differences between different treatments by least significant difference test (*p* < 0 0.05).

### 3.12 Comprehensive Analysis of Mantel Test, Principal Component and Random Forest Model Analysis

As shown in Figure S1A, RDS content was highly significantly positively correlated with ALP, ST, TC, and ΔH_ret_, and significantly positively correlated with SSG, RC, PV, TV, FV, BD, T_O_, T_P_, ΔH_gel_, %R, SP, SO, BV, and *λ*_max_. RS showed significant positive correlations with ALP, ST, SSG, PV, TV, FV, BD, T_O_, TC, Δ_Hgel_, ΔH_ret_, SP, SO and BV. MSG and 1045/1022 ratio were negatively correlated with SDS (Figure S1A). PCA (Figure S1B) revealed that PC1 and PC2 explained 46.0% and 17.3% of variance, respectively. ΔHret, RDS, SSG, BV, RC, %R, T_o_, ΔH_gel_ and T_c_ were core indicators for PC1, PC2 variation was mainly explained by MSG and 1045/1022. Random forest (Figure S1C–E) showed that ALP, TS, RC, SSG and ΔH_ret_ contributed most to RDS variation. SDS were primarily associated with BD, MSG, ΔH_gel_ and AGS. RS was mainly determined by RC, ΔH_ret_, PV and FV.

## 4 Discussion

### 4.1 Effects of Foliar Application of ICE6 on the Starch Content, Structure and Particle Size of Waxy Maize

As an iron complex with a core porphyrin structure similar to chlorophyll, ICE6 acts as a photosensitizer to enhance chlorophyll's light energy capture efficiency, thereby improving leaf photosynthetic rate and photosynthetic nitrogen use efficiency (Nasar et al. 2022). This improvement in photosynthetic performance provides a sufficient carbon source for grain carbohydrate accumulation, directly driving the increase in soluble sugar, total starch, and amylopectin contents. In this study, T5 and T6 treatments increased total starch and amylopectin contents. These changes are attributed to ICE6 optimization of key enzyme activities of carbon metabolism, which promotes soluble sugar synthesis and their directional conversion into amylopectin (Jian et al. 2025), consistent with previous conclusions on regulator‐mediated leaf carbohydrate metabolism (Cao et al. 2016). Notably, amylose content showed no significant differences among all treatments, indicating that ICE6 exerts selective regulation on starch synthesis pathways, mainly influencing amylopectin synthesis with limited effects on amylose synthesis (Nasar et al. 2022).

The morphology, dispersibility and crystalline structure of starch granules are the structural basis determining their functional properties, and photosynthetic carbon supply adequacy directly regulates starch granule development (Nasar et al. 2022). In this study, ICE6 treatments maintained the basic starch granule morphologies and caused significant differences in granule integrity, surface state, particle size and dispersibility; T5 produced the largest, nearly spherical and plump granules with pristine surfaces and perfect monodispersity. T6 induced obvious surface indentations but achieved the optimal crystalline ordered structure with the most abundant and bright polarized light signals. Further observation revealed that starch samples exhibited a typical A‐type crystal structure, consistent with previous research on waxy maize starch (Chen et al. 2024), but decreased starch relative crystallinity. Fourier transform infrared (FTIR) spectroscopy analysis further revealed that ICE6 enhanced starch surface orderliness and reduced surface disorder, with starch crystallinity positively correlated with the 1022/995 cm^−1^ absorbance ratio (Chen et al. 2014). The decrease in relative crystallinity and short‐range order indicated that ICE6 may interfere with hydrogen bonding interactions between starch molecular chains (Liu, Jiang, et al. 2021).

The effect of ICE6 on starch particle volume distribution resulted from its concentration dependent regulation of key starch synthesis enzyme activities. In this study, low ICE6 concentrations stimulated ADP‐glucose pyrophosphorylase activity at the initial stage of starch synthesis, promoting more polymerization center formation and thus significantly increasing small starch granule volume proportion in T3; with increasing ICE6 concentration, starch synthase and starch branching enzyme activities were optimized, and their synergistic action drove small granule growth and maturation into medium granules (Nakamura 2002), leading to a reduction in the small granule proportion under T5 and T6 treatments, respectively, and a significant increase in medium granule proportion. Meanwhile, ICE6 significantly reduced large granule volume proportion, possibly due to the high concentrations regulating starch synthase activity balance and limiting excessive granule growth (Liu, Jiang, et al. 2021). In terms of average particle size, T2–T6 showed significant reduction compared with T1, indicating that ICE6 optimizes particle size distribution by promoting a shift to medium granules with superior functional properties, which is closely associated with improved internal crystalline ordered structure (Yang et al. 2019).

### 4.2 Effects of Foliar Application of ICE6 on the Starch Pasting, Thermodynamic, and In Vitro Digestive Characteristics of Waxy Maize

The effects of ICE6 on starch pasting properties are closely linked to altered granule size distribution and crystalline structure. In this study, this gelatinization viscosity enhancement was mainly due to the reduced volume proportion of large starch granules—structurally intact large granules are difficult to disrupt (Wang et al. 2021), and their reduction facilitates starch granule gelatinization and swelling. Additionally, the optimized starch granule structure under ICE6 enhanced water absorption and swelling capacity, promoting the formation of a stable gelatinization and further improving pasting properties (Liu, Jiang, et al. 2021). ICE6 is unfavorable for paste viscosity recovery, consistent with research on common buckwheat starch (Gao et al. 2022) and closely related to ICE6's inhibition of starch retrogradation, a property with important application value in low‐retrogradation food processing. In this study, ΔH_gel_ and ΔH_ret_ showed a reduction in T2–T6 treatments, which is directly related to decreased relative crystallinity; a higher ΔH_gel_ value indicates higher crystallinity and molecular organization (Zhou et al. 2020), and the reduction fully demonstrates that ICE6 weakens crystalline structure stability by interfering with hydrogen bonding, making starch easier to swell and disrupt during heating (Liu, Jiang, et al. 2021). Meanwhile, %R showed significant reduction in T2–T6 treatments, indicating that ICE6 effectively inhibits starch retrogradation, possibly by promoting post‐gelatinization starch molecular chain association to form a steric hindrance effect, impeding the reformation of ordered double‐helical structures or crystalline regions during cooling (Liang et al. 2025).

Foliar application of ICE6 reshaped the multi scale structure of waxy maize starch, leading to significant concentration dependent changes in its in vitro digestive characteristics. Among them, T4, T5, and T6 treatments showed a significant reduction in RDS content compared with T1. In contrast, SDS content increased in T2, T4, T5, and T6 relative to T1, indicating that ICE6 had a consistent promoting effect on SDS accumulation without obvious dose‐dependent enhancement in the tested range. RS, the fraction resisting enzymatic hydrolysis in the small intestine (Zheng et al. 2020), showed a similar upward trend to SDS, with T4 and T5 achieving significant increases. This variation of decreased RDS and increased SDS and RS induced by ICE6 is consistent with the pattern of heat‐moisture treated purple rice flour (Chuwech et al. 2023), proving that ICE6‐mediated foliar application is an effective agronomic measure to regulate starch digestive properties via structural modification in waxy maize production.

Mantel test further quantified the significant regulatory relationships between starch physicochemical properties and in vitro digestion traits, RDS had a highly significant positive correlation with amylopectin content, starch crystallinity, gelatinization temperature and ΔH_ret_, and a significant positive correlation with SSG volume proportion, pasting viscosity indicators, swelling power and solubility. The positive correlation between RDS and SSG proportion was due to the larger specific surface area of small granules providing more binding sites for digestive enzymes to accelerate hydrolysis (Liu et al. 2015), higher pasting viscosity and enthalpy values mean sufficient structural disruption of starch granules during gelatinization, facilitating enzyme penetration and decomposition (Jane et al. 1999; Cooke and Gidley 1992). RS had a significant positive correlation with relative crystallinity, PV, FV and ΔH_ret_, as short‐range ordered structures form a tight hydrogen‐bonding network and physical encapsulation hinders digestive enzyme access (Cai and Shi 2010; Zou et al. 2022), thus increasing RS content. Notably, SDS showed no significant correlation with any measured physicochemical property, as its digestion is jointly regulated by multiple factors, whose complex interactions mask the correlation with individual indicators. PCA of 30 measured parameters showed PC1 and PC2 cumulatively explained 63.3% of total variation, with ΔH_ret_, RDS, SSG proportion, crystallinity and %R as core PC1 indicators, and MSG proportion and FTIR ratios as main PC2 factors, confirming particle size distribution, gelatinization and thermodynamic properties as the key regulators of digestive characteristics. In summary, foliar application of ICE6 modulates starch synthesis and structural characteristics by enhancing leaf photosynthesis and optimizing carbon metabolism, with a prominent concentration effect and T5 treatment as the optimal application concentration. ICE6 promotes the accumulation of total starch and amylopectin, and also reshapes the multi‐scale structure of starch granule, which in turn optimizes starch pasting, thermodynamic and swelling properties, and ultimately regulates the in vitro digestive characteristics by altering the enzymatic hydrolysis efficiency of starch granules.

## 5 Conclusions

This study investigated the effects of foliar application of ICE6 on the physicochemical properties and in vitro digestibility of waxy maize starch. Foliar application of ICE6 at 90 g ha^−1^ improved starch quality by integrating multi‐scale structural optimization, enhanced physicochemical properties, and favorable digestibility modulation. Specifically, this treatment improved granule morphology, dispersion, and regularity, increased the volume proportion of medium‐sized granules, and elevated amylopectin content and surface order, reduced relative crystallinity. These structural enhancements translated into superior physicochemical properties, including increased swelling power, solubility, pasting viscosity, gelatinization enthalpy, which collectively optimized the starch digestibility profile by reducing rapidly digestible starch content. Notably, ICE6 is a novel plant growth regulator whose foliar application aligns with the green agriculture development strategy in the Beijing–Tianjin–Hebei region, as it reduces reliance on conventional fertilizers and promotes sustainable cultivation practices. Therefore, this study provides a theoretical reference for high‐quality waxy maize starch production and supports the formulation of regional cultivation practices oriented towards green and high‐quality development of fresh‐eating waxy maize.

## Author Contributions

**Linxiao Liu:** investigation, data curation, writing – original draft. Meng Zhang: methodology, investigation. **Yujing Zhang:** software, investigation. **Mengyu Li:** methodology, visualization. **Xiangling Li:** writing – review and editing, funding acquisition.

## Funding

This work was supported by the Hebei Province Central Guidance Fund for Local Scientific and Technological Development Projects (246Z6401G).

## Ethics Statement

The authors have nothing to report.

## Conflicts of Interest

The authors declare no conflicts of interest.

## Supporting information


**Figure S1:** Correlation analysis (A) and Principal components analysis (B) between starch physicochemical properties and in vitro digestibility. Random forest model determining critical variables impact RDS (C), SDS (D) and RS (E) of waxy maize.
**Table S1:** Effects of foliar application of iron chlorine e6 on the starch pasting properties of waxy maize.
**Table S2:** Effects of foliar application of iron chlorine e6 on the starch thermal properties of waxy maize.

## Data Availability

The data that support the findings of this study are available from the corresponding author upon reasonable request.
