# Correction to “Effects of Foliar Application of Iron Chlorine e6 on the Starch Physicochemical Properties and In Vitro Digestibility of Waxy Maize”

**DOI:** 10.1002/fsn3.72291

**Published:** 2026-08-25

Liu L. , M. Zhang , Y. Zhang , M. Li , and X. Li . 2026. “Effects of Foliar Application of Iron Chlorine e6 on the Starch Physicochemical Properties and In Vitro Digestibility of Waxy Maize.” Food Science & Nutrition 14: e71978. 10.1002/fsn3.71978.42255695 PMC13238892

In the Acknowledgments and Funding sections, the statement “This research was supported by the Hebei Province Central Guidance Fund for Local Scientific and Technological Development Projects (246Z6401G)” was incorrect. This should have read “This research was supported by the Local Science and Technology Development Fund Project Guided by the Central Government (246Z6401G).”

In Figure 3B (b), the significance letter above the T4 bar was incorrect. The corrected figure is provided below.

**FIGURE 3:**
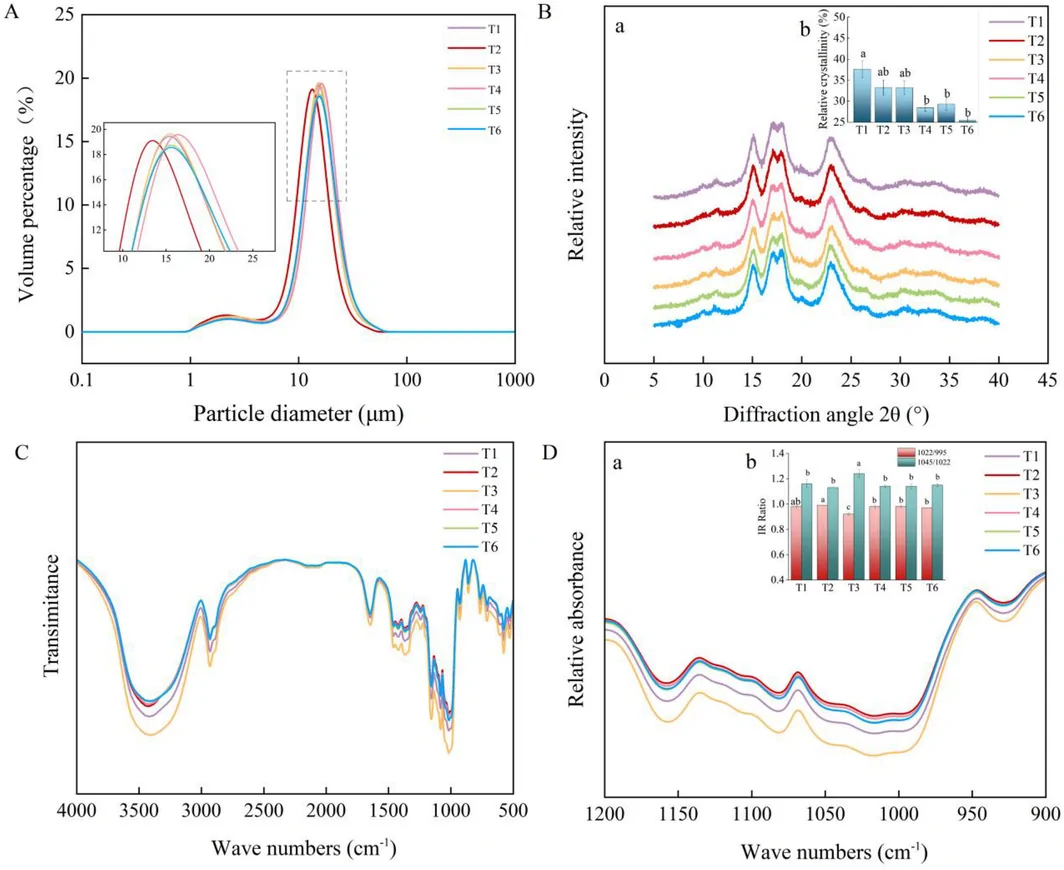
Effects of foliar application of iron chlorine e6 on the starch granule volume distribution (A), X‐ray diffraction (XRD) patterns (a) and (b) relative crystallinity (B), Fourier‐transform infrared (FTIR) full spectrum (C), and FTIR spectra in the 900−1200 cm^−1^ region (a) and IR absorbance ratios of 1022/995 and 1045/1022 cm^−1^ (b) reflecting short‐range ordered structure (D). T1, T2, T3, T4, T5, and T6 denoted as the iron chlorine e6 application rates at 0, 22.5, 45, 67.5, 90, and 180 g ha^−1^, respectively. Different lowercase letters indicate significant differences among treatments (*p* < 0.05).

In Table 1, the Total starch and Amylopectin were incorrect. The corrected table is provided below.

**TABLE 1:** Effects of foliar application of iron chlorine e6 on the grain nutritional quality of waxy maize.

Treatment	Sugar (%)	Total starch (%)	Amylopectin (%)	Amylose (%)	Amylopectin proportion (%)
T1	5.71 ± 0.56d	42.92 ± 0.94b	42.77 ± 0.94bc	0.13 ± 0.01a	99.55 ± 0.02a
T2	6.43 ± 0.57c	43.28 ± 0.84b	43.13 ± 0.83b	0.12 ± 0.01a	99.59 ± 0.01a
T3	7.11 ± 0.42b	43.63 ± 0.42b	43.50 ± 0.44b	0.12 ± 0.01a	99.60 ± 0.03a
T4	8.40 ± 0.42a	44.23 ± 0.67b	44.07 ± 0.67b	0.12 ± 0.03a	99.59 ± 0.06a
T5	8.60 ± 0.35a	46.48 ± 0.28a	46.36 ± 0.24a	0.10 ± 0.04a	99.53 ± 0.09a
T6	8.20 ± 0.51a	46.72 ± 0.95a	46.58 ± 0.94a	0.09 ± 0.02a	99.55 ± 0.02a

*Note*: T1, T2, T3, T4, T5, and T6 denoted as the iron chlorine e6 application rates at 0, 22.5, 45, 67.5, 90, and 180 g ha^−1^, respectively. Data are means of three replications. Means with no letter in common indicate significant differences between different treatments by least significant difference test (*p* < 0.05).

We apologize for these errors.

