# Supplementary material for: Effects of Foliar Application of Iron Chlorine e6 on the Starch Physicochemical Properties and In Vitro Digestibility of Waxy Maize
Source: Food Sci Nutr. 2026 Jun 4;14(6):e71978. doi: 10.1002/fsn3.71978 (PMC13238892; doi:10.1002/fsn3.71978)
Supplement: Supplementary file 1 — Figure S1: Correlation analysis (A) and Principal components analysis (B) between starch physicochemical properties and in vitro digestibility. Random forest model determining critical variables impact RDS (C), SDS (D) and RS (E) of waxy maize. Table S1: Effects of foliar application of iron chlorine e6 on the starch pasting properties of waxy maize. Table S2: Effects of foliar application of iron chlorine e6 on the starch thermal properties of waxy maize. [file FSN3-14-e71978-s001.doc]

TABLE S1 Effects of foliar application of iron chlorine e6 on the starch pasting properties of waxy maize

| Treatment | PV(cP) | TV(cP) | FV(cP) | BD(cP) | SB(cP) |
| --- | --- | --- | --- | --- | --- |
| T1 | 1340.33±49d | 533.00±21e | 637.33±10e | 807.33±31c | 104.33±11a |
| T2 | 1532.00±29c | 612.00±34d | 674.00±27d | 920.00±18b | 62.00±8bc |
| T3 | 1557.33±39c | 676.67±15c | 785.67±16bc | 880.67±24b | 109.00±29a |
| T4 | 1596.00±116bc | 664.33±56cd | 761.67±22c | 931.67±59b | 97.33±36ab |
| T5 | 1689.33±22b | 760.67±14b | 801.33±10b | 928.67±20b | 40.67±6c |
| T6 | 1872.00±6a | 874.67±16a | 972.33±16a | 997.33±15a | 97.67±5ab |

Note： T1, T2, T3, T4, T5 and T6 denoted as the iron chlorine e6 application rates at 0, 22.5, 45, 67.5, 90 and 180 g ha⁻¹, respectively. Data are means of three replications. Means with no letter in common indicate significant differences between regulator by least significant difference test (p < 0.05).

TABLE S2 Effects of foliar application of iron chlorine e6 on the starch thermal properties of waxy maize

| Treatment | To(℃) | Tp(℃) | Tc(℃) | ΔH_gel_(J/g) | ΔH_ret_（J/g） | % R (%) |
| --- | --- | --- | --- | --- | --- | --- |
| T1 | 72.36±0.95a | 73.63±1.43b | 77.95±2.26abc | 4.62±0.26a | 1.79±0.12a | 38.79±4.65a |
| T2 | 74.60±0.54a | 76.53±0.62a | 79.48±0.4a | 3.43±0.37b | 1.13±0.08b | 32.94±1.66ab |
| T3 | 71.51±1.85a | 75.73±0.92a | 79.88±0.38a | 3.93±0.27b | 1.26±0.09b | 32.10±0.19ab |
| T4 | 74.65±4.4a | 75.65±0.74a | 78.23±0.04ab | 3.07±0.34c | 0.77±0.18c | 24.86±3.18bc |
| T5 | 65.68±2.89b | 72.64±0.63b | 76.49±0.49bc | 3.60±0.23b | 0.74±0.09cd | 20.62±3.67c |
| T6 | 63.48±2.13b | 73.27±1.13b | 76.16±0.14c | 2.36±0.81d | 0.54±0.05d | 24.99±8.84bc |

Note: T1, T2, T3, T4, T5 and T6 denoted as the iron chlorine e6 application rates at 0, 22.5, 45, 67.5, 90 and 180 g ha⁻¹, respectively. Data are means of three replications. Means with no letter in common indicate significant differences between regulator by least significant difference test (p < 0.05).


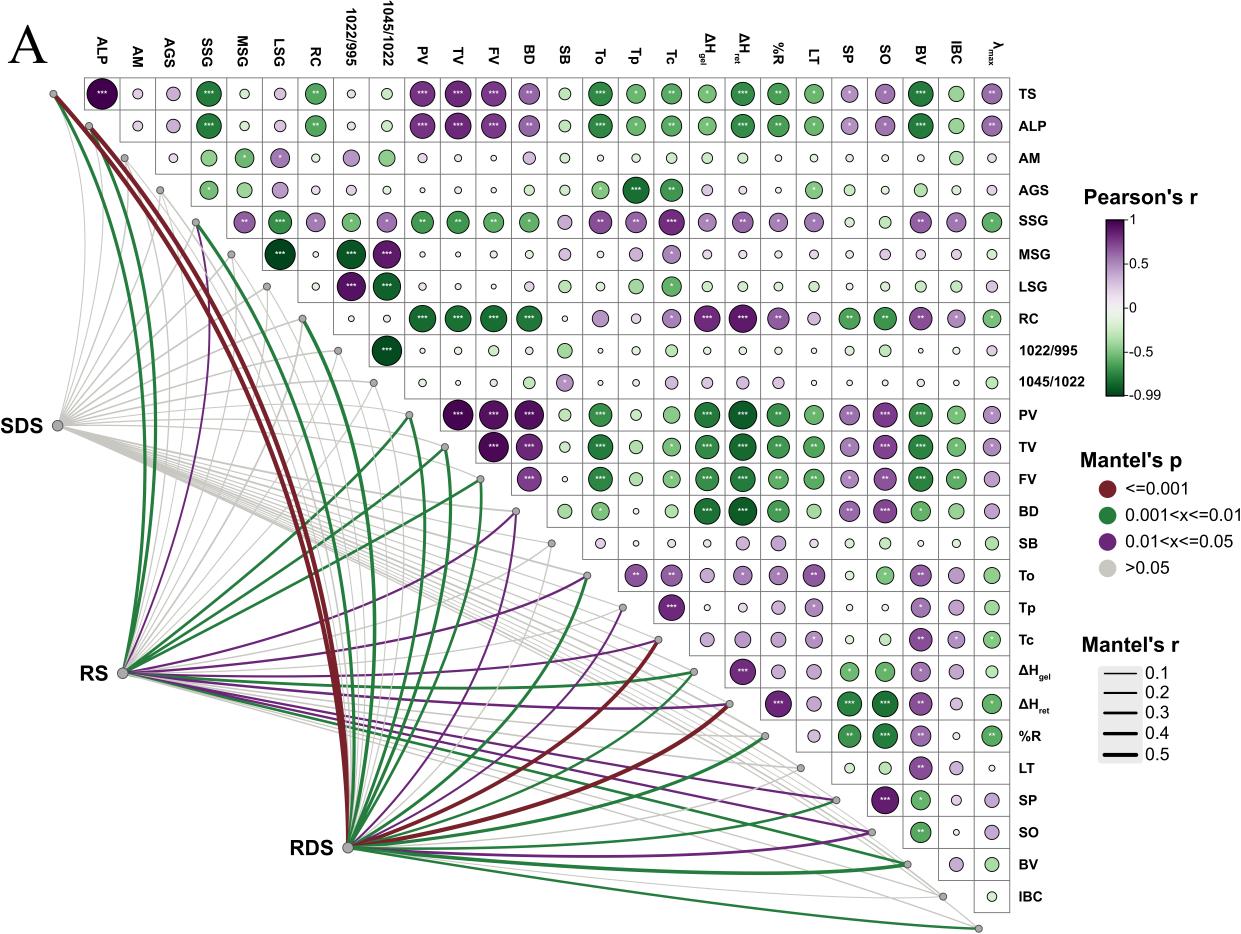


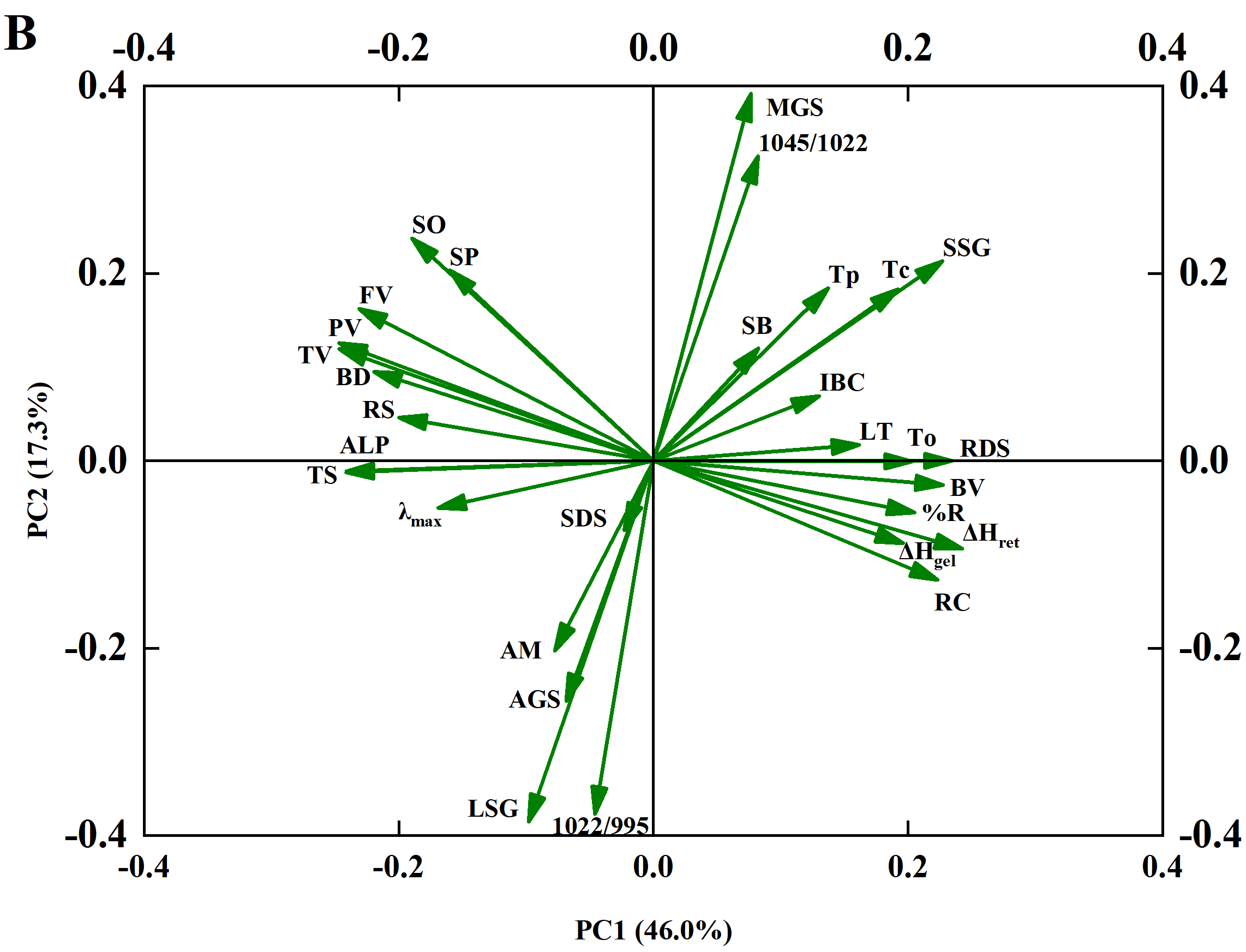

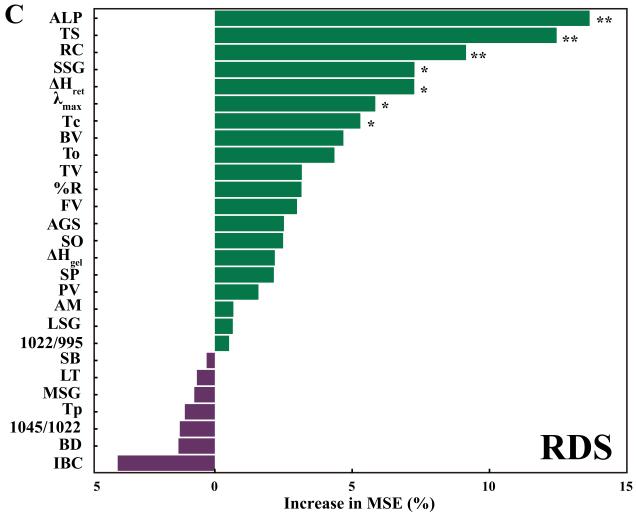


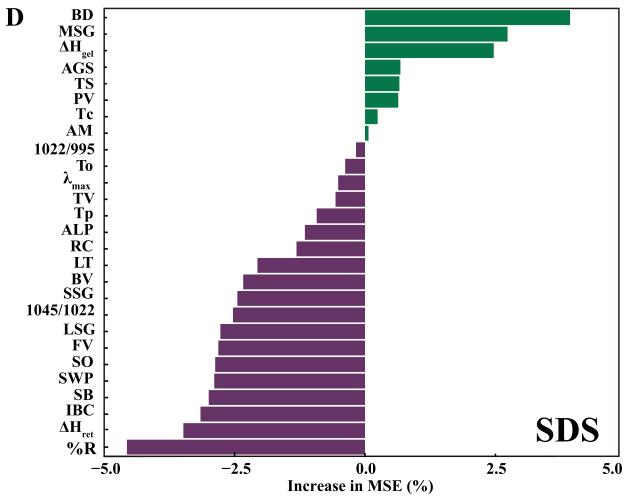

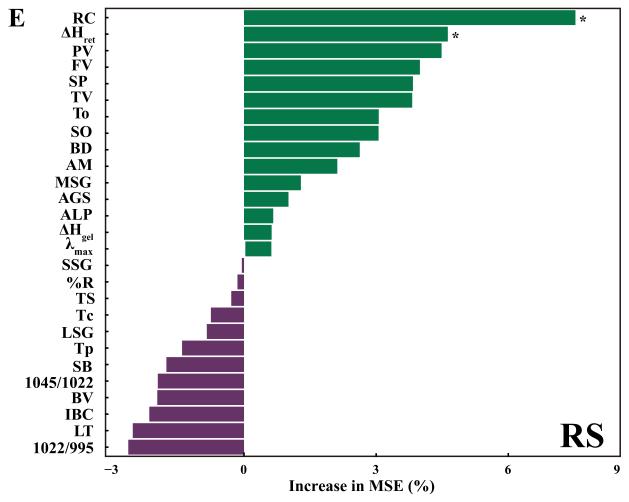


**FIGURE S1** Correlation analysis (A) and Principal components analysis (B) between starch physicochemical properties and in vitro digestibility. Random forest model determining critical variables impact RDS(C), SDS(D) and RS (E) of waxy maize. %IncMSE indicates the increase in the mean squared error (MSE) of prediction RDS SDS and RS after permuting this variable. Significant effects are depicted with P<0.05, : P<0.01; Yellow: positive correlation; Blue: negative correlation; The darker the color, the stronger the correlation.TS, total starch; ALP, Amylopection; AM, Amylose; AGS, Average starch granule size; SSG, percentage of small starch granule(d < 5 µm); MSG, percentage of medium starch granule (5 µm ≤ d ≤ 15 µm); LSG, percentage of large starch granule (d >15 µm); 1022/995, the ratio of absorbance 1022/995 cm^-1^; 1045/1022, the ratio of absorbance 1045/1022 cm^-1^; RC, Relative crystallinity; LT, Light transmittance; SP, Swelling power; SO, solubility; BV: Blue value; IBC, Iodine binding capacity; λ_max_, maximum absorption wavelength; PV, peak viscosity; TV, trough viscosity; FV, final viscosity; BD, breakdown; SB, setback; To, onset temperature; Tp, peak temperature; Tc, completion temperature; △H_gel_, phase transition enthalpy of original starch; ΔH_ret_, phase transition enthalpy of regeneration starch; %R, regeneration rate. RDS, rapidly digestible starch; SDS, slowly digestible starch; RS, resistant starch
